# 715 Ocular Complications of Facial Burns in the Pediatric Population

**DOI:** 10.1093/jbcr/irad045.191

**Published:** 2023-05-15

**Authors:** Abigail Teitelbaum, Annmarie Craig, Elika Ridelman, Lisa Bohra, Christina Shanti, Sharmila Segar

**Affiliations:** Wayne State University School of medicine, Detroit, Michigan; Wayne State University School of Medicine, Detroit, Michigan; Department of Surgery, Division of Pediatric Surgery, Children's Hospital of Michigan/Wayne State University School of Medicine, Detroit, Michigan; Children's Hospital of Michigan, West Bloomfield, Michigan; Department of Surgery, Division of Pediatric Surgery, Children's Hospital of Michigan/Wayne State University School of Medicine, Detroit, Michigan; Kresge Eye Institute, Wayne State University, Northville, Michigan; Wayne State University School of medicine, Detroit, Michigan; Wayne State University School of Medicine, Detroit, Michigan; Department of Surgery, Division of Pediatric Surgery, Children's Hospital of Michigan/Wayne State University School of Medicine, Detroit, Michigan; Children's Hospital of Michigan, West Bloomfield, Michigan; Department of Surgery, Division of Pediatric Surgery, Children's Hospital of Michigan/Wayne State University School of Medicine, Detroit, Michigan; Kresge Eye Institute, Wayne State University, Northville, Michigan; Wayne State University School of medicine, Detroit, Michigan; Wayne State University School of Medicine, Detroit, Michigan; Department of Surgery, Division of Pediatric Surgery, Children's Hospital of Michigan/Wayne State University School of Medicine, Detroit, Michigan; Children's Hospital of Michigan, West Bloomfield, Michigan; Department of Surgery, Division of Pediatric Surgery, Children's Hospital of Michigan/Wayne State University School of Medicine, Detroit, Michigan; Kresge Eye Institute, Wayne State University, Northville, Michigan; Wayne State University School of medicine, Detroit, Michigan; Wayne State University School of Medicine, Detroit, Michigan; Department of Surgery, Division of Pediatric Surgery, Children's Hospital of Michigan/Wayne State University School of Medicine, Detroit, Michigan; Children's Hospital of Michigan, West Bloomfield, Michigan; Department of Surgery, Division of Pediatric Surgery, Children's Hospital of Michigan/Wayne State University School of Medicine, Detroit, Michigan; Kresge Eye Institute, Wayne State University, Northville, Michigan

## Abstract

**Introduction:**

Pediatric burns commonly involve the face and periocular areas, with a risk of impairing vision. The aim of this study is to characterize ocular injuries in burn patients and identify the patients at most risk of ocular complications.

**Methods:**

This study is a retrospective review within a single academic, urban pediatric burn center. All burn patients under 18 years of age admitted from January 2010 to December 2020 with periorbital or ocular involvement were included. Variables analyzed included patient demographics, burn characteristics, presence of ophthalmology consultation, ocular exam findings, follow up time period, and early and late ocular complications.

**Results:**

In the study period, 2,781 patients were admitted to our burn center, 300 of whom had facial burns involving the eyes and/or eyelids. Etiologies of burn injuries were as follows: 112 (37.5%) scald, 80 (26.8%) flame, 35 (11.7%) contact, 31 (10.4%) chemical, 28 (9.4%) grease, and 13 (4.3%) friction. Overall, 70.9% of patients with ocular burns received an ophthalmology consult. Of these patients, 61.5% had periorbital swelling and 39.8% had corneal injuries. Of the 207 patients who were followed as inpatients by ophthalmology, only 61 (29.5%) presented for a follow-up visit, as recommended, 6 of which had serious ocular sequelae including ectropion, entropion, symblepharon, and corneal decompensation, and 4 of whom had a firework-related injury.

**Conclusions:**

While burns involving the ocular surface and eyelid margins are infrequent overall, they can lead to long-term sequelae. Ophthalmology consultation is critical, particularly in the setting of chemical and firework-related injury. As ocular and periocular burns can cause immediate as well as delayed sequelae, ophthalmologic evaluation is a key component of assessment.

**Applicability of Research to Practice:**

This paper characterizes ocular injuries in pediatric patients with facial burn injuries and identifies patients most at risk for ocular complications. We believe this will be valuable for many burn researchers and clinicians in order to better identify patients who could benefit from ophthalmologic evaluation and those at risk for both immediate as well as delayed sequelae from periocular burns.

